# The intertwined metabolism during symbiotic nitrogen fixation elucidated by metabolic modelling

**DOI:** 10.1038/s41598-018-30884-x

**Published:** 2018-08-21

**Authors:** Thomas Pfau, Nils Christian, Shyam K. Masakapalli, Lee J. Sweetlove, Mark G. Poolman, Oliver Ebenhöh

**Affiliations:** 1 Institute of Complex Systems and Mathematical Biology, University of Aberdeen, Aberdeen, UK; 2 School of Basic Sciences, Indian Institute of Technology Mandi, Mandi, India; 3 Department of Plant Sciences, University of Oxford, Oxford, UK; 4 Department Biological and Medical Sciences, Oxford Brookes University, Oxford, UK; 5 Institute of Quantitative and Theoretical Biology, Cluster of Excellence on Plant Sciences CEPLAS, Heinrich-Heine-University Düsseldorf, Düsseldorf, Germany; 6 Life Sciences Research Unit, University of Luxembourg, Belvaux, Luxembourg

## Abstract

Genome-scale metabolic network models can be used for various analyses including the prediction of metabolic responses to changes in the environment. Legumes are well known for their rhizobial symbiosis that introduces nitrogen into the global nutrient cycle. Here, we describe a fully compartmentalised, mass and charge-balanced, genome-scale model of the clover *Medicago truncatula*, which has been adopted as a model organism for legumes. We employed flux balance analysis to demonstrate that the network is capable of producing biomass components in experimentally observed proportions, during day and night. By connecting the plant model to a model of its rhizobial symbiont, *Sinorhizobium meliloti*, we were able to investigate the effects of the symbiosis on metabolic fluxes and plant growth and could demonstrate how oxygen availability influences metabolic exchanges between plant and symbiont, thus elucidating potential benefits of inter organism amino acid cycling. We thus provide a modelling framework, in which the interlinked metabolism of plants and nodules can be studied from a theoretical perspective.

## Introduction

Nitrogen belongs to the elements which are absolutely crucial for life, because it is contained in all amino acids and many other essential biomolecules. Whereas nitrogen is the most abundant element in the earth’s atmosphere, most of it is present in the inert form of nitrogen gas (N_2_) constituting approximately 80% of the total atmosphere. Therefore, despite its high abundance, nitrogen is often a limiting factor for growth in plants. To overcome this in agriculture, large amounts of nitrogen are applied to the soil in the form of artificial fertilisers to promote plant growth. Before the industrial revolution, farmers applied a crop rotation scheme to their fields and left the field barren or planted it with beans or peas every few years to let the soil ‘recover’. We now know that this strategy re-introduces bio-available nitrogen into the soil. This is due to ability of the planted crops (beans or peas) or plants quickly occupying barren fields, such as clovers, to form a nitrogen fixing symbiosis with rhizobia. Rhizobia carry a gene for the highly oxygen sensitive nitrogenase enzyme, which catalyses the reduction of atmospheric nitrogen to ammonium, and the plants provide the symbiont with an environment protecting it from damaging oxygen. In this way atmospheric nitrogen is introduced into the global organic nutrient cycle. Simultaneously the nitrogenase requires large amounts of energy and reductants to catalyse nitrogen fixation. This has led to the development of a tightly regulated oxygen supply for oxidative phosphorylation by the plant to the symbiont^1,2^. This symbiosis has seen the most attention and is best understood in plants of the Fabacaea (or Leguminosae) family, to which beans, peas and clovers belong^3–7^. In recent years, *Medicago truncatula* has become a model plant for the legume-rhizobia symbiosis^8^. While there is a plethora of rhizobial strains, only a limited number interact which each host^9,10^. *Sinorhizobium meliloti* is a well-studied symbiont of *M*. *truncatula*^11^ and therefore an optimal organism to model this symbiosis. Low nitrogen availability will trigger the recruitment of rhizobia to the plant roots and initiate the nodulation, in which rhizobia invade the plant root and nodules are formed. In these nodules, the rhizobia are taken up by plant cells, surrounded by a membrane and differentiate into bacteroids. Upon completing differentiation they begin fixing nitrogen, which is made available to the plant primarily in the reduced form of ammonium. In return, the plant provides organic acids as nutrients to the rhizobia^4,12^. In addition there is evidence that amino acid cycling is essential for nitrogen fixation at least in some rhizobial strains^13,14^. Obviously, the mutual dependence of the metabolism of plant and rhizobium is crucial for this symbiotic interaction. During the establishment of the nodules, the metabolic fluxes in both plant and rhizobium change drastically^15^.

To explore, understand and analyse the changes in the metabolic fluxes during nodulation, computer simulations have become increasingly established in modern biology research. For this, detailed high quality metabolic network reconstructions have to be established. Whereas there exist now over a hundred^16,17^ genome-scale metabolic network reconstructions for a wide range of organisms, and a number of steps in the development of these models can now efficiently be automated^18,19^, the reconstruction of genome-scale metabolic networks still involves numerous manual curation steps and therefore is very time-consuming^20^. However, even before a reconstruction is finished and established simulation techniques, such as flux balance analysis (FBA) can be employed, the building process itself provides considerable insight into the metabolic capabilities of the investigated organism and allows to refine its genome annotation. Once a highly curated metabolic network reconstruction is established, it presents a theoretical framework allowing to query the system and understand its functional properties. The numerous possible theoretical investigations^21^ are for example useful to help understanding the structure and regulation of metabolic networks^22,23^, identify essential genes^24^, predict putative drug targets^25^ or support engineering of novel pathways^26^ producing desired compounds of technological or economic interest. In the plant sciences, genome-scale models with different degrees of accuracy and completeness exist for the model unicellular green alga *Chlamydomonas reinhardtii*^27^, the model species *Arabidopsis thaliana*^22,28^, rice *Oryza sativa*^29^ and others^30,31^. While initially plant models were unicellular representations of metabolism, not distinguishing between different tissues, recent efforts tend to refine these models and generate multi-tissue representations^32^. While the network structure and catalysed reactions are now fairly well established and can be extracted using the genome annotation, most enzyme kinetics are still unknown. This makes the use of ODE based kinetic models impossible on genome-scale networks, as thousands of parameters would have to be estimated. FBA is a modelling technique that addresses this issue by imposing a steady state assumption on the fluxes, indicating that there is no change of internal metabolite concentrations in the network^33^. FBA further imposes an objective that the model should optimise (e.g. minimal effort to produce a compound or maximisation of biomass^34^), which yields a unique optimal value. Thus the network can be investigated for the effects of a changing environment without the need for extensive parameter fitting and optimisation. Unfortunately, the flux distribution itself (in contrast to the optimal value) is not necessarily unique in FBA^35^. This can however be addressed by using minimisation of the overall quadratic flux, which represents a solution with efficient enzyme usage, for a given optimal FBA value, at which point the flux distribution becomes unique.

In this paper, we focus on the presentation of a highly curated genome-scale metabolic reconstruction of *M*. *truncatula* and provide an analysis of general metabolic properties of its biochemical reaction network. For our detailed and curated reconstruction, which contains 1636 reactions in 7 compartments, we have developed an extended approach to assign metabolic reactions to sub-cellular compartments. Our approach integrates not only sequence and proteomics derived localisation information^36,37^ but also integrates known importers and exporters, thus taking advantage of an additional source of information. This information is integrated using the concept of network extension introduced in Christian *et al*.^38^. All reactions in our reconstruction were manually curated to achieve complete mass- and charge-balance as suggested in Thiele and Palsson^39^. This important property of a network allows for a system wide prediction of fluxes of protons and other charged particles over cellular membranes. To our knowledge, we present the first plant metabolic model, for which charge balancing is ensured on genome scale, while this was previously only achieved for small subsystems^22^ or core metabolism^40,41^. Charge balancing over membranes is a key prerequisite to realistically describe electron transfer chains required for ATP biosynthesis. Compartmentalisation allows for a more precise distinction between the function and necessity of isozymes which are present in multiple compartments. Omitting this information would allow the model to use reactions for which substrates are not available, because they are not transported between compartments. This would make the prediction of potentially lethal mutations less reliable. We further refined the model by integrating tissue specific information to better represent the distribution of metabolism to root and shoot of the plant.

We discuss insights gained from the reconstruction process itself and their consequences on the annotation of genes with previously unknown functions and the refinement of annotations of previously insufficiently annotated genes. We experimentally obtained tissue specific biomass data of *M*. *truncatula*, thus providing for the first time a system wide in-depth biomass composition for this plant. To investigate the interaction between plant and microbe, this highly curated plant model was connected to a model of *Sinorhizobium meliloti*, derived from the MetaCyc database^19^, and we investigated the effect of nitrogen fixation and symbiosis on the plant model. The presented model aims at simulating the nitrogen fixing state of the symbiosis and does not reflect the process of forming the symbiosis. We further addressed the question which fluxes might be most restrictive for nitrogen fixation and investigated the effect of small alterations of oxygen supply on the symbiotic nitrogen fixation capacity. The model presented in this paper provides an important resource for researchers in plant sciences in general and for studying plant-rhizobia interactions in particular. Our simulations help to interpret and understand responses of plant metabolism, which include rearrangements of metabolic fluxes and changes in energy and reductant requirements, as a result of changes in nitrogen availability.

## Results

### Experimental biomass Analysis leads to suggested gene annotation update

During the biomass analysis of *M*. *truncatula* (details can be found in Supplementary Table 5) we found pinitol in the plant. When trying to determine the biosynthetic pathway for this substance we found that the enzyme encoded by gene *MTR_4g038440* (Entrez Gene ID: 11446905), currently annotated as a caffeic acid 3-O-methyltransferase, is very similar (87% sequence identity) to the inositol methyltransferase from *Glycine max* (Entrez Gene ID: 100812768). Since we could not find other candidates for this biosynthetic step, we expect that this enzyme might indeed be mis-annotated and would suggest a revision of its annotation.

### A multi-tissue model of *Medicago truncatula*

**Figure 1:**
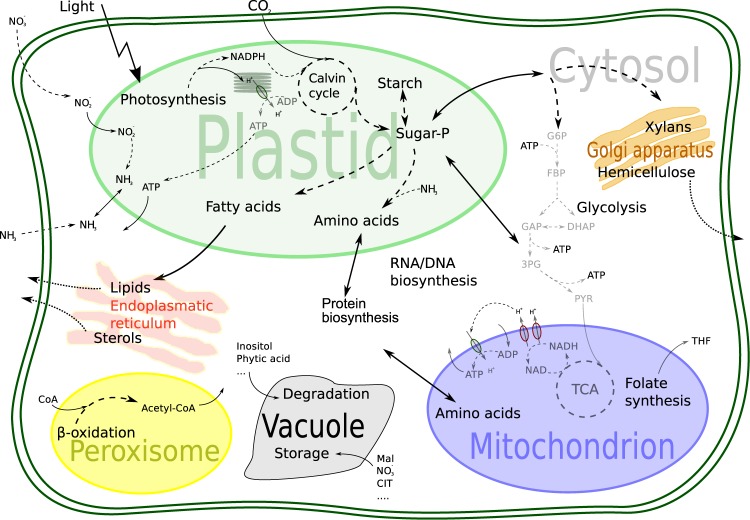
Schematic overview of the metabolic processes and compartments included in the genome-scale model of *M*. *truncatula*.

We created a genome scale model of *Medicago truncatula* encompassing 3403 genes coding for 2909 reactions in 8 compartments using 2780 metabolites. An overview of the model is provided in Fig. 1 and a more detailed description of this network can be found in the Supplementary Text 7.

This genome-scale model encompasses our knowledge of biochemical reactions catalysed by enzymes, which are encoded in the genome along with additional necessary reactions to fulfil known functions. However, it is unable to describe processes in a particular cell type or part of the organism, as no such separation is yet included. We therefore constructed a multi-tissue model of *Medicago truncatula* using data from Benedito *et al*.^42^ employing the FASTCORE^43^ algorithm (see Materials and Methods for details).

The shoot tissue of this model was represented by 969 reactions associated with a total of 1824 genes acting on 860 metabolites and containing 126 internal transporters. The root tissue in turn consists of 958 reactions associated with a total of 1804 genes acting on 846 metabolites and including 122 transporters. These submodels were connected by 32 inter-tissue transporters and a combined biomass reaction, with the inter-tissue transporters derived from the literature^32,44,45^. The large reduction can partly be explained by many reactions in secondary metabolism which, in the current model formulation, are unable to carry flux, because specific exchange reactions have not been added, and because concentrations of those secondary metabolites are too small to be detected during biomass analysis. In total, the consistent part of the *Medicago truncatula* network contained only 1303 reactions, which are able to carry non-zero flux with the current configuration of exchange reactions, while all remaining reactions can be activated if additional exporters are included. The two-tissue model was able to sustain growth during day (using light as energy source and photosynthesis for carbon) and night (with starch as energy and carbon source). In addition, both ammonium and nitrate can be used as nitrogen sources to support growth under day and night conditions.

### Computational analysis

#### Differences between day and night

We investigated the model under day and night conditions to determine differences in network usage under these conditions. *In silico* knockout experiments predict that during night conditions, 236 reactions are essential (including 26 transporters). Under phototrophic growth, 243 reactions are essential (25 transporters). In total, 232 reactions are essential under both conditions. Starch degrading reactions are essential only under night conditions, while those involved in the Calvin cycle and photosynthesis are essential only in light conditions. Interestingly, the additional transporter predicted as essential under dark conditions is the maltose exporter in the chloroplast envelope (*mex*). However, *mex* mutants are viable, albeit with a reduced growth and starch excess phenotype^46^. The reason that the model predicts this transporter as essential is that, in the strict mathematical sense, no flux distribution exists which fulfils the stationarity condition for maltose. Therefore, the prediction can be understood in terms of the mathematical model formulation, and the predicted phenotype (maltose accumulation) is one of the key characteristics of the *mex* mutant.

#### Nitrogen metabolism in the two tissue model

**Figure 2:**
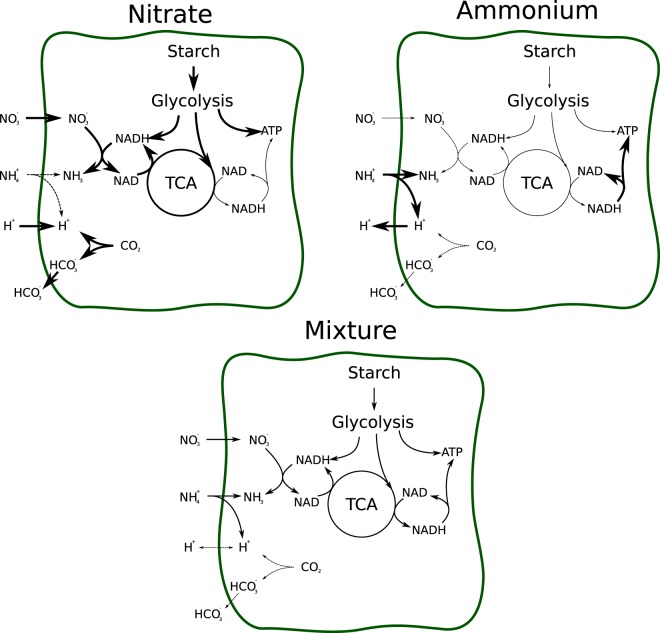
Fluxes changing with alternating nitrogen sources. Nitrate nutrition requires large amounts of reductants produced by the TCA and leads to an export of negative charges (via HCO3^−^). Ammonium nutrition is energetically cheaper, and allows the use of reductants for ATP synthesis, while simultaneously leads to a export of protons from the plant.

Since *M*. *truncatula* is a model organism for symbiotic nitrogen fixation, the effects of different forms of available nitrogen are of high relevance. The main sources for nitrogen are ammonium and nitrate. However, supplying both of these nitrogen sources and directly applying either flux minimisation or carbon usage minimisation would lead to the trivial result that the model selects ammonium as the source, because it is ‘cheaper’ in terms of energy and reductant (see Supplementary Text 7 and Supplemetal Dataset 1). To simulate a transition, we therefore restricted the amount of nitrogen available to the plant and scanned over a range of different ammonium vs nitrate compositions. To calculate the fluxes, we fixed the growth rate to 0.1 g/gDW per day and minimised the sum of the squares of all fluxes in the system. Figure 2 depicts different conditions of pure ammonium, mixed and pure nitrate provision.

As expected the amount of required starch drops with increasing ammonium availability. At the same time, the TCA activity is reduced, as less reductant is required for nitrate reduction. Simultaneously the mitochondrial ATPase activity increases, using parts of the freed reductant. The model also indicates soil acidification during ammonium nutrition due to a high export of protons. Under mixed nutrition, this export is balanced since charge and protons are balanced when a equimolar amount of nitrate and ammonium are imported. Under pure nitrate nutrition, this exchange is actually reversed, and protons are taken up while simultaneously HCO3^−^ is released. While a large fraction of these exported charges will in nature be balanced by the uptake of either positive or negative ions^47^, this computational results still nicely illustrates and at least partially explains the acidifying properties of ammonium nutrition in contrast to nitrate nutrition^48^.

#### Symbiotic nitrogen fixation

**Figure 3:**
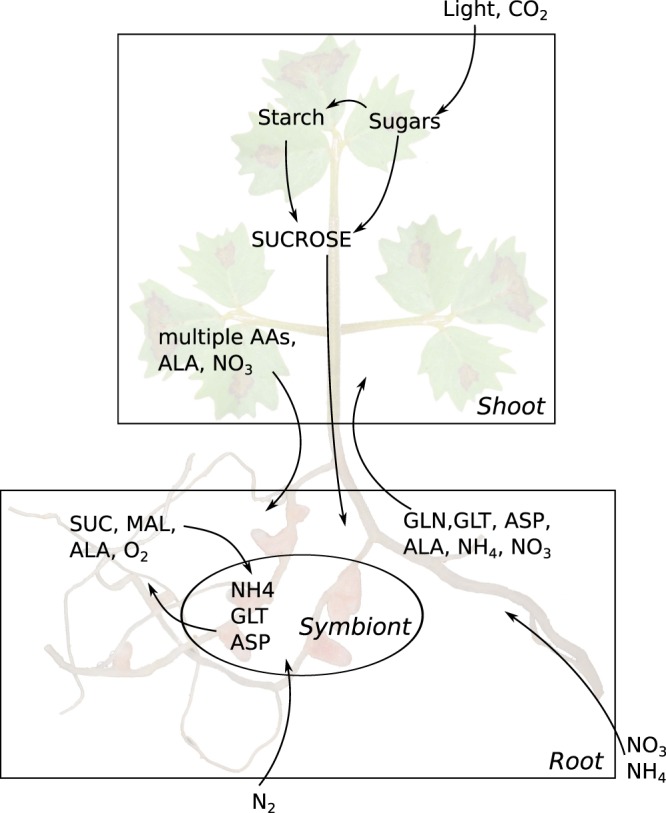
Layout of the two-tissue model combined with its symbiotic partner. The most important exchanges between the tissues and between plant and symbiont are shown. Original figure (here in background) by Ninjatacoshell (https://commons.wikimedia.org/wiki/File:Medicago_truncatula_A20_root_nodules.JPG), “Medicago truncatula A20 root nodules”. Removed the black background and used as part of the background of the combined work by Thomas Pfau, https://creativecommons.org/licenses/by-sa/3.0/legalcode.

We first used the combined model (see the schematic in Fig. 3) to test under which conditions a symbiotic association with *S*. *meliloti* is beneficial to the host *M*. *truncatula*.

**Figure 4:**
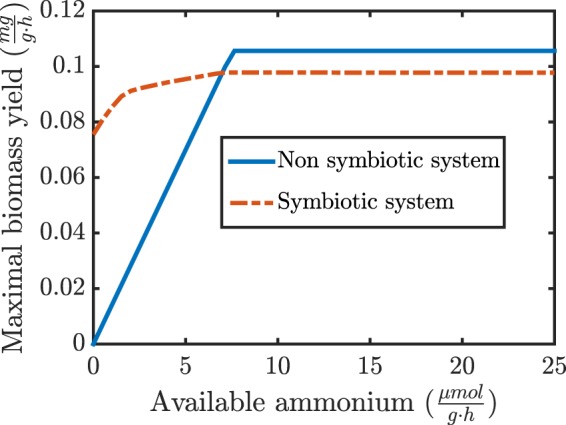
Comparison of the maximal growth of the symbiotic and non-symbiotic system. While the symbiotic system can grow without available ammonium by fixing nitrogen, this advantage necessitates a maintenance of the rhizobial symbiont, which reduces the energy available to the plant, leading to a lower maximal growth when sufficient ammonium is available.

For this, we compared the maximal predicted growth rate of the models with and without nitrogen fixing symbiont, for different external ammonium concentrations (see Fig. 4).

Clearly, the growth rate is slightly lower for the symbiotic system if sufficient nitrogen is available. This can be explained by the additional energy requirement to produce organic carbon to support the symbiont. However, under low nitrogen availability (in the form of nitrate or ammonium) the ability to fix nitrogen by the rhizobium allows for plant growth even without any external nitrogen source present. Thus, this combined model can explain and to some extent quantify under which conditions a symbiotic relationship is advantageous.

There are multiple propositions in the literature concerning the transport of nitrogen from the rhizobium to the plant^13,49,50^. An initial investigation showed that the amount of oxygen available to the bacteroids is the main limiting factor for the amount of nitrogen that can be fixed. This is expected, because the nitrogenase requires large amounts of energy, which is most efficiently provided by oxidative phosphorylation. However, while a higher oxygen concentration provides more energy to the bacteroid, the nitrogenase complex becomes irreversibly inactivated if oxygen concentrations are too high^51^. Therefore the plant actively regulates the oxygen supply using leghemoglobin, ensuring sufficient oxygen for fixation, but minimising detrimental effects to the nitrogenase activity^2^. As such, it is interesting to see what effects slightly changing oxygen amounts would have on the symbiosis. While an increased potential biomass production is the most obvious effect of a better oxygen supply it is also interesting to investigate how the exchange fluxes between bacteroid and plant are affected if the amount of fixed nitrogen remains constant, but oxygen concentrations varies. For this, we fixed the growth rate of the plant to 80% of the maximal growth rate (determined above), and systematically varied the oxygen availability for the bacteroid and calculated the fluxes based on the assumption that enzymes are used in a maximally efficient way (see Methods).

**Figure 5:**
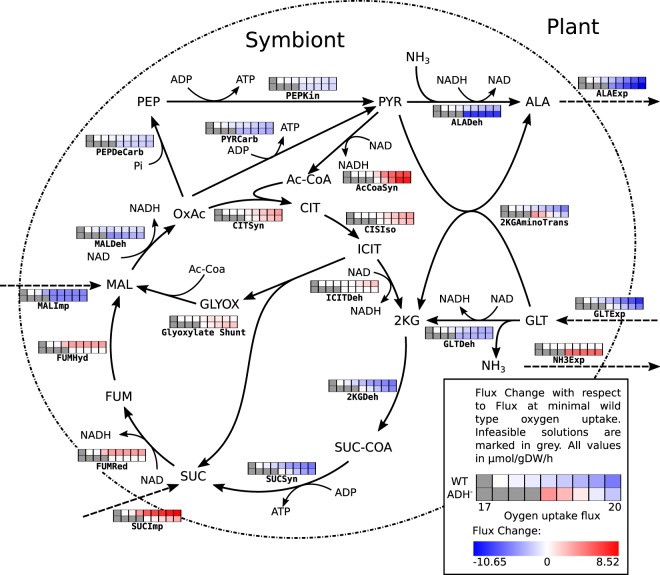
Changes of fluxes for changing oxygen uptake by the symbiont. Grey values indicate no feasible solution (i.e. insufficient oxygen for sufficient nitrogen fixation). The upper row corresponds to wild type solutions while the lower row represents alanine dehydrogenase knockout. NH3 export is only active in the mutated strain, while all nitrogen is exported as alanine in the wild type simulation (marked in orange, as the export does not change with oxygen in both conditions). With increasing oxygen availability, succinate is preferentially used as carbon source as it provides additional reductant compared to Malate. Amino acid shuttling is reduced with increasing oxygen in both conditions, due to the increased potential of complete respiration of carbon. Under oxygen limiting conditions, pyruvate is generated instead of citrate, which can either be converted to alanine (wild type), consuming reductant, or can be used as acceptor for the amino group from glutamate.

The model predicts extensive amino acid cycling between the plant and the symbiont (see Fig. 5) in agreement with earlier findings^13^. Alanine is predicted to be the only nitrogen-containing export product. The amino group of glutamate is transferred by transaminases to pyruvate, yielding the export product alanine and ketoglutarate. The latter is fed into the TCA cycle, where one ATP is generated by succinyl-CoA synthase. Newly fixed nitrogen is exported as alanine, which is synthesised *de novo* from pyruvate by alanine dehydrogenase. Pyruvate in turn is produced by reverse action of pyruvate carboxylase. The standard Gibbs free energy of reaction of pyruvate carboxylase is Δ*G*^0^ = −1.37 kcal/mol (according to MetaCyc), but due to the comparatively large amounts of dicarboxylic acids and the high demand for ATP it appears plausible that the equilibrium can easily be shifted to pyruvate. The imported carbon source required for pyruvate synthesis depends strongly on the available oxygen. For low oxygen supply, the capacity of the respiratory chain is limited and import of malate minimises the amount of produced NADH. With increasing oxygen availability, ATP production by oxidative phosphorylation is increasingly efficient. This explains the observed switch from malate to succinate uptake, because introduction of succinate into the TCA cycle provides one additional reductant as compared to malate. Therefore succinate is only used when sufficient oxygen is available (or when no malate is provided).

In our simulation alanine was the only nitrogen export product from the rhizobia. However, there have been experiments in which alanine dehydrogenase was knocked out, showing that *de novo* alanine synthesis is not required for symbiotic growth^52,53^. Our model allows a rapid reproduction of this experiment *in silico*. Our simulations (Fig. 5 and Supplementary Text 7) show, even under these conditions, a substantial export of alanine, which is directly cycled against glutamate. However, in the knock-out simulation, nitrogen is exported from the symbiont in the form of ammonia (increased flux compared to WT in Fig. 5), which is subsequently assimilated in the plant. In the knock-out conditions, the minimum amount of required oxygen increases (additional grey boxes for low Oxygen supply), which corresponds to a slightly decreased growth, a phenomenon observed by Allaway *et al*.^52^ but not by Kumar *et al*.^53^. Interestingly, the knock-out simulation does not show any uptake of dicarboxylic acids at minimal oxygen concentrations but relies solely on the use of glutamate as carbon source. This can be explained by the fact that using glutamate as carbon source allows, as described above, the use of succinyl-CoA synthase to regenerate ATP, while simultaneously producing reductants. Thus, without the ability of a *de novo* alanine production, an optimal ratio of ATP to reductant is provided by pure glutamate uptake. With additional oxygen, the use of succinate as reductant donor and oxidative phosphorylation as ATP source becomes more efficient.

## Discussion

We have developed a genome scale compartmentalised model for the clover *Medicago truncatula*, a model plant for the legume–rhizobia symbiosis. We have carefully verified that our model is thermodynamically feasible, that all relevant intermediates can be replenished, and all reactions are balanced with respect to both mass and charge, which allows the interpretation of results regarding the effect of different types of nitrogen nutrition on the environmental pH. The reconstruction process was useful in its own right, as it resulted in the putative suggestion of a novel gene annotation.

However, new annotations are not the primary objective for building detailed genome-scale metabolic models. More importantly, a genome-scale model provides a theoretical framework in which experimental observations can be interpreted and understood. Computational analyses allow to query the model, assess its capabilities, and enable novel interpretations of experimental observations in a theoretical context. An interesting observation is derived by our calculation of the theoretically maximal conversion rate of starch-derived carbon into biomass at night. Considering only energy and redox requirements for the formation of biomass, a remarkably high percentage of starch carbon (92% for growth on ammonium, 79% for growth on nitrate) can be converted into biomass. These numbers show the theoretical maximal conversion rate of starch carbon into biomass carbon which will likely not reflect the real situation. However, these deliberations do allow an estimation of the non growth associated maintenance costs based on measurements of starch usage and respiration like those by Pyl *et al*.^54^. Experiments performed in *A*. *thaliana*, in which respiration and starch degradation rates were carefully measured throughout the night^54^, showed that at least 45% of the starch is respired. This value is still more than twice as high as the predicted minimum of 21% for the growth on nitrate. Moreover, Pyl *et al*.^54^ showed that the ratio of respired starch-derived carbon is highly dependent on the night temperature. The lowest value of 45% is observed for low (12 °C) temperatures, while for nightly temperatures of 24 °C (which was the same as the applied temperature during the day), the ratio of respired carbon increased to 75%. Estimating from our calculations that around 20% of carbon contained in starch need to be respired to build biomass, this allows to conclude that between 25% and 55% of starch-derived carbon is respired during the night for maintenance, depending on temperature and probably other external factors. This is in good agreement with previous findings of maintenance requirements of around 40% by Williams *et al*.^55^. This comparison is similar to the approach by Cheung *et al*.^56^ to estimate the non growth-related maintenance energy. However, while they based their findings on ATP fluxes in a cell culture, we base our estimates on starch consumption in planta. The derived energy is required for processes which are not directly related to growth and not included in our model, such as degradation and repolymerisation of proteins and mRNA, as well as transport processes across intracellular membranes or phosphorylation in regulatory pathways. But why the ratio is so strongly dependent on the ambient temperature remains unclear. To further understand the detailed requirements for maintenance energy, it will be necessary to formulate mathematical models of maintenance requirements based on experimental measurements of protein and mRNA turnover and intracellular transport^57–59^. The results of such models would allow to define further constraints on genome-scale metabolic models as the one presented here, and lead to a more profound understanding of the regulation of metabolic fluxes responding to environmental changes.

Using only a model of the host of the symbiosis it is already possible to make some investigations into the symbiotic properties. The dominating factor in the symbiosis is the transfer of nitrogen between host and symbiont. We therefore first investigated the response of metabolic fluxes in the host to changing nitrogen sources. If both nitrate and ammonium are abundant, the model predicts an exclusive uptake of ammonium, because integration into amino acids requires considerably less reductants and energy when compared to nitrate. This result is in agreement with the experimental observation that nitrate uptake is inhibited when ammonium is available^60^. Thus, in an evolutionary context, our model suggests that the reason for this inhibition is an increased energetic efficiency. Simultaneously proton export is observed which is in accordance with soil acidification when ammonium fertilisers are used^48^. If ammonium becomes limiting, the modelled fluxes change gradually to an increased uptake of nitrate, which is accompanied by a higher energy and reductant demand that is met by increased respiration during the night. The balancing of charges, using both ammonium and nitrate, might contribute to the observation that the presence of ammonium in many plants^61^ inhibits nitrate uptake but does not turn it off completely, while from an energetic consideration, nitrate should not be used at all. At the same time, we have to be aware of the limitations of FBA when interpreting computational results simulating changes in environmental pH. In particular, ionic barriers commonly employed by plants cannot be modelled in FBA.

By combining the plant network with a rhizobial symbiont, we were able to illustrate the evolutionary advantages in undergoing a symbiosis and to simultaneously show that it can come at a cost when formed in a rich environment. We could demonstrate that the observations of amino acid cycling and alanine as nitrogen carrier fit into a paradigm of efficiency. Further, we were able to suggest that the reason for the use of alanine as export product lies in the ability to remove some surplus reductant from the bacteroid system, but that it is not strictly necessary for symbiotic growth. However, it is interesting that even when alanine dehydrogenase is knocked out, we observed the export of alanine (now exchanged with glutamate), which supports the hypothesis that alanine is a major export product in planta, even without being the nitrogen carrier.

Rhizobia represent only one component of a highly complex and dynamic microbial community associated with plant roots^62^. In natural environments this community may contain beneficial microbes, delivering nutrients extracted from the soil to the plant in exchange for reduced carbon, and parasites, living on dead or live plant material. In principle, our approach to combine several network models is also applicable to more complex interaction networks^63^. However, the bottleneck is often our limited knowledge on the exchanged metabolites between the different players. Moreover, by increasing the complexity of the network, also the model complexity and concomitantly the possible model solutions increase dramatically. We therefore view our approach, focusing on a well-investigated model system, in which only two organisms interact, as a first step towards studying more complex interaction networks.

## Materials and Methods

For a more extensive description of the Materials and Methods, we refer the reader to the Supplemental Text 7, only a short description will be provided here.

### Model Reconstruction

The plant model is based on version 3.5v5 of the *Medicago truncatula* genome annotation^64^ and was reconstructed using PathwayTools^65^ along with the MedicCyc database^66^. For compartmentalisation, data from multiple databases (TAIR for homologies^67^, SUBA for localisations^68^) and recent studies^69,70^ in *M*. *truncatula* where used for initial localisation. Further localisations were assigned using an approach by Christian *et al*. to make all compartments consistent^38^. All seeds and targets used can be found in Supplemental Dataset 2. Transporters were added according to information from the literature^71–74^ and where they were necessary to allow compounds which could be produced in the uncompartmentalised network to be still producible in the compartmented network. Reaction directionalities were adjusted to avoid thermodynamically infeasible cycles.

### Generation of a two tissue shoot-root Network

The two tissue model for root and shoot was created using the FASTCORE algorithm^43^ and gene expression data from Benedito *et al*.^42^. This was done by duplicating the model and connecting the tissues similar to an earlier approach^32^. For all connecting transporters see Supplemental Dataset 3. Finally, models able to grow in day and night conditions using either ammonium or nitrate as substrate were generated from the combined model and all reactions present in one of the networks were added to the final model. Data for ATP Maintenance and growth rate were extracted from De Vries and Penning^75^ and Lötscher *et al*.^76^, respectively.

### Building a symbiotic system

A network for *Sinorhizobium meliloti* 1021 was extracted from MetaCyc. The model was curated to achieve mass and charge balance of all reactions and to allow it to produce most biomass components from an *E*.*coli* biomass definition^77^ in order to ensure a generally viable model. The model was connected to the plant root submodel using exchange reactions present in the literature (for Reviews see e.g^12,15,78^. and a list of used transporters can be found in Supplemental Dataset 4). Links between rhizobium fluxes and plant fluxes were generated based on existing literature information^79–82^.

### Network analysis

Our simulations are based on the underlying assumption of a metabolic quasi steady state used in Flux Balance Analysis^83^, where internal concentrations do not change over time:

1 $${\bf{S}}\cdot {\bf{v}}=0,$$

With S being the stoichiometric matrix with the position (*i*, *j*) representing the coefficient of the i-th metabolite in the j-th reaction, and *v* being the vector of fluxes (i.e. reaction activities). Further we in general assume that the organisms have evolved to perform whichever task they are required with a maximal efficiency. This is represented in our simulations by minimisation of the flux through the network, which corresponds to a minimal necessity for enzyme synthesis. We commonly minimise the quadratic flux values^84^. In contrast to minimising the simple sum of fluxes, this yields a unique solution^34,85^:

2 $$min\sum _{i}^{n}\,{({v}_{i})}^{2}$$

Additional constraints based on literature (e.g. maintenance ATP, maximal photon availability etc.) are introduced. To test multiple conditions, we solve this system for multiple fixed values of target reactions and compare the fluxes for these ranges. Examples of these scans can be found in the Supplemental Text 7.

### Availability of scripts and reconstruction

Matlab scripts that reproduce the scans in Supplemental Text 7 underlying Figs 2 and 5 along with the CPLEX interface for ScrumPy are available online at https://github.com/sysbiolux/MedicagoScripts. The reconstruction is also available on the github site and in Supplementary Dataset 5.

### Biomass composition determination

Biomass composition was determined according to adapted protocols by Williams *et al*.^86^ and Masakapalli *et al*.^87^. Plants were grown following the hydroponic culture protocol from the *Medicago truncatula* Handbook^88^. More details can be found in Supplemental Text 7. Data for biomass distribution from^89^ and^90^ was used. Cell wall composition was extracted from^91,92^, and^93^. The lipid composition was based on^94^. DNA GC Content was derived from^95^.

## Electronic supplementary material

Supplementary Dataset 1

Supplementary Dataset 2

Supplementary Dataset 3

Supplementary Dataset 4

Supplementary Dataset 5

Supplementary Dataset 6

Supplementary Text S7
