# Supplementary material for: The intertwined metabolism during symbiotic nitrogen fixation elucidated by metabolic modelling
Source: Sci Rep. 2018 Aug 21;8:12504. doi: 10.1038/s41598-018-30884-x (PMC6104047; doi:10.1038/s41598-018-30884-x)
Supplement: Supplementary file 7 — Supplementary Text S7 [file 41598_2018_30884_MOESM7_ESM.pdf]

Short Title: Metabolic Model of the Legume-Rhizobium Symbiosis

The intertwined metabolism during symbiotic nitrogen fixation elucidated by metabolic modelling - Supplementary Results

Thomas Pfau[1,6], Nils Christian[1], Shyam K. Masakapalli[2], Lee J. Sweetlove[3], Mark G. Poolman[4], and Oliver Ebenhöh[1,5]

[1]Institute of Complex Systems and Mathematical Biology, University of Aberdeen

[2]School of Basic Sciences, Indian Institute of Technology Mandi

[3]Department of Plant Sciences, University of Oxford

[4]Department Biological and Medical Sciences, Oxford Brookes University

[5]Institute for Quantitative and Theoretical Biology, Cluster of Excellence on Plant Sciences CEPLAS, Heinrich-Heine-University Düsseldorf

[6]Life Science Research Unit, University of Luxembourg

June 18, 2018

1 Supplemental Results

Properties of the *Medicago truncatula* metabolic network Starting from the annotated genome version 3.5v5 [1] and the biochemical information in the MedicCyc (v1.0) [2] and BioCyc [3] databases, we first built a database using the PathwayTools program [4].

Based on this refined database, we next built a metabolic network model without information about intracellular compartments (see Methods). After an initial manual curation to fill gaps (see Gap Filling), this network contained 1636 reactions.

To get an initial overview of the biochemical processes involved, we have grouped these reactions into the different categories of metabolism, based on the BioCyc categorisation. Figure 1 shows the numbers of reactions assigned to the different MetaCyc pathway categories. While a majority of reactions (1117) are involved in biosynthesis pathways, only 294 are involved in degradation processes. Several reactions are present in both categories, because some processes are involved in both biosynthesis and degradation pathways.

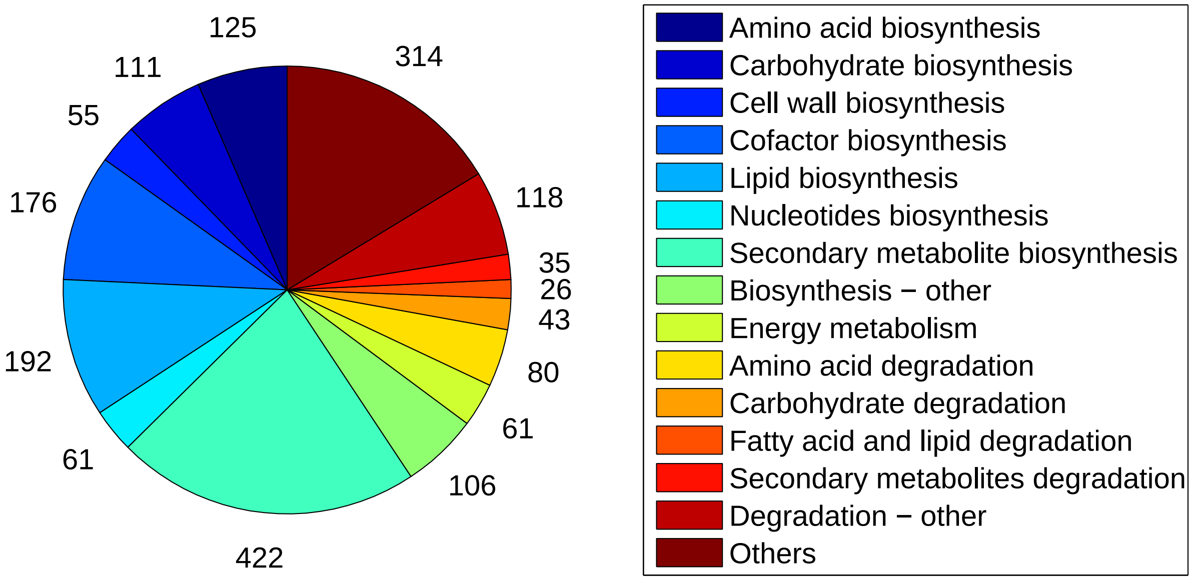

Figure 1: Categorisation of the reactions in the model based on MetaCyc pathway categories. 1117 reactions are involved in biosynthesis processes and 293 reactions are involved in degradation pathways, with 113 reactions being present in both categories. There are several biosynthetic reactions involved in multiple biosynthesis processes and a single degradation reaction involved in multiple degradation processes. The category 'Others' comprises all reactions present in the network, which are not transporters, importers or exporters and are either not assigned to any pathway or not included in any of the categories shown.

To fully compartmentalise our model and assign the 1636 reactions to the 7 compartments (cytosol, mitochondria, plastid, peroxisome, enodplasmic reticulum, vacuole and Golgi), we applied the procedure detailed in the Methods section. In short, first experimental evidence for sub-cellular localisation [5, 6, 7] was used to assign the corresponding reactions to a compartment. Subsequently, the network extension method [8] was applied to add reactions to the different compartments, ensuring essential biochemical functions associated with each compartment. All reactions that remained unlocalised after this step were then assigned to the cytosol. A final manual curation step assured that metabolites producible in the uncompartmentalised network were still producible in the compartmentalised network in at least one compartment. Figure 2 gives an overview of the compartmentalisation process and the assignment of the reactions to the different compartments. The large increase in the number of reactions associated with the ER in the final curation step results from a manual assignment of various sterol biosynthesis pathways to the ER [9].

Roughly a third (572) of the reactions are present in more than one compartment leading to a total of 2526 reactions in the model. For most of these reactions their multiple localisation was inferred based on homology to Arabidopsis [7] or evidence from proteomic studies [5, 6] while few were predicted by the network extension algorithm. The compartments are connected by a total of 271 transporters taken from the literature [10, 11, 12, 13] or inferred in the final gap filling step (see Materials and Methods). The model reactions connect 1370 distinct chemical species, of which 701 are present in more than one compartment, resulting in a total of 2780

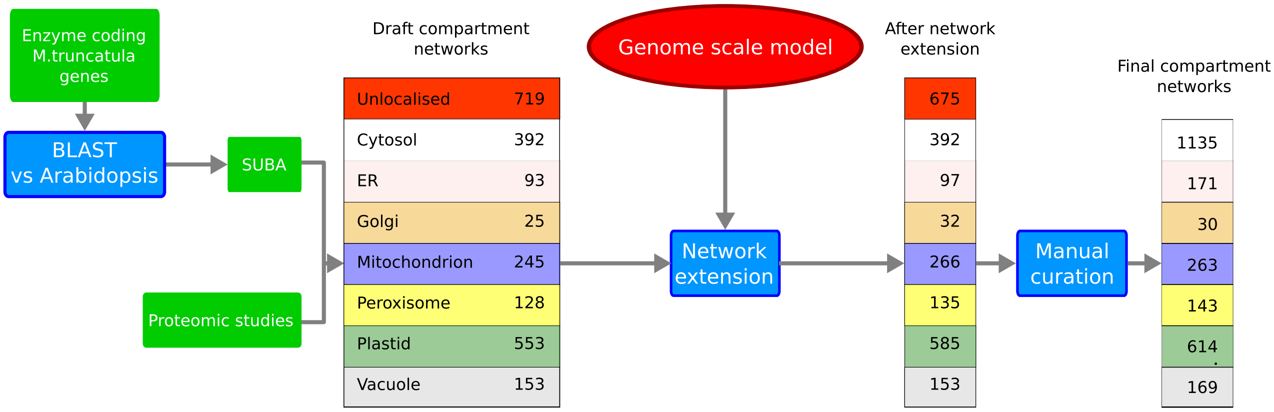

Figure 2: Process of compartment localisation. Assigning compartments to reactions was performed in three steps. The first step comprises a BLAST search for Arabidopsis homologues and obtaining proteomic data [5, 6]. In a second step, network extension [8] is applied to ensure that every organelle can perform key functions. The uncompartmentalised genome-scale model serves as a reference network. In a third step, it is ensured by manual curation that all compounds are producible which were producible in the uncompartmentalised network.

unique metabolic species in the model. Genetic evidence is present for over 70% of all non transport reactions.

1.1 Computational Analysis

Network self-consistency During cellular growth, all intermediate metabolites are diluted and have to be replenished. These metabolites are not included in the biomass, but the necessity to reproduce them results from the fact that cells grow and divide [14, 15]. We therefore first tested whether the model is self-consistent in the sense that all metabolites can be produced from the nutrients using constraint-based modelling (see Methods). We found that 1161 of the 1370 metabolites could be produced. The remaining 209 metabolites fall into three main classes.

The first class, containing 121 metabolites, includes those substances which form conserved quantities. Examples are the acetyl carrier proteins linked to fatty acids, macromolecular redox carriers, such as ferredoxins, and tRNAs, which are necessary for the incorporation of amino acids into proteins. Most of these macromolecules are small proteins and RNA molecules which are implicitly contained in the biomass as part of the protein and nucleic acid fractions. Since the model can produce biomass (see below), biosynthesis is possible for over 86% of the compounds in this class. The remaining are small molecules, which cannot be biosynthesized because they are themselves needed for their own biosynthesis. An example is quinate, for which no biosynthetic route is present in MetaCyc, where it is only included as a substrate for degradation. However it is used in one of two routes from coumaroyl-CoA to caffeoyl-CoA, in which it is needed as a carrier compound.

The second class contains 65 metabolites, which are not connected to the remaining network. This group contains many of the biotin biosynthesis pathway intermediates (6 compounds). The reason why this pathway is

disconnected is that it is unknown how the pimeloic acid moiety necessary for biotin biosynthesis is synthesised in plants. Most of the other metabolites (37) in this class are produced by specific enzymes, but no pathway for the production of their precursors is known.

The third class consists of 23 metabolites, which result from the degradation of modified macromolecules (e. g. polyfructofuranose), or which are present only in a degradation pathway for xenobiotics (e. g. cyanate). This phenomenon of metabolites which can only be 'produced' by degradation was discussed in detail earlier [8].

Differences between day and night cont'd Of the essential metabolic reactions (excluding transporters) mentioned for day and night biomass production over 90% have genetic evidence. Our knockout simulations further revealed an additional set of 16 and 18 reactions for growth in the dark and light, respectively, which are essential in the sense that they must be present in at least one compartment, but in the compartmentalised model they have been assigned to several compartments. The two additional reactions for which at least one isozyme has to be present in light conditions are the triose isomerase reaction and phosphoglycerate kinase, which are both necessary to utilise Calvin cycle-derived triose phosphates. Using the objective to minimise the total flux through the system while producing a given amount of biomass (see Methods), we found that a set of 430 (dark) and 423 (light) reactions, of which 72 are transporters, are sufficient to produce all biomass components.

Energy requirements for biomass precursors During night, plants use the stored starch as carbon and energy source. To investigate the nightly energy metabolism, it is interesting to know how much energy and reductant are minimally required to build essential building blocks, such as nucleotides, amino acids and organic acids. The theoretically predicted minimal energy and reductant requirements for selected compounds are depicted in Fig. 3, when using as nitrogen source either ammonium (clear bars) or nitrate (shaded bars). Obviously, the requirements depend critically on the nitrogen source. Growing on ammonium, most nucleotides and some amino acids, along with some organic acids and sugars can theoretically be directly produced from starch without additional ATP obtained through respiration (a full list including the reductant and energy costs is provided in the Supplementary Table S2). In fact, some of the compounds can be synthesised from starch while even producing a small surplus of energy equivalents. In contrast, with nitrate as nitrogen source, the synthesis of all amino acids and nucleotides requires additional ATP and NAD(P)H, for which some of the starch needs to be respired. This is not surprising because the reduction of nitrate to ammonia requires four reducing equivalents. Using nitrate exclusively, only organic acids are still producible from starch without additional energy demands. Because they do not contain nitrogen, their requirements are independent on the nitrogen source. Our calculations are in agreement with earlier observations that heterotrophic cell cultures of *A. thaliana* grown on nitrate tend to produce higher levels of organic acids and sugars and lower levels of

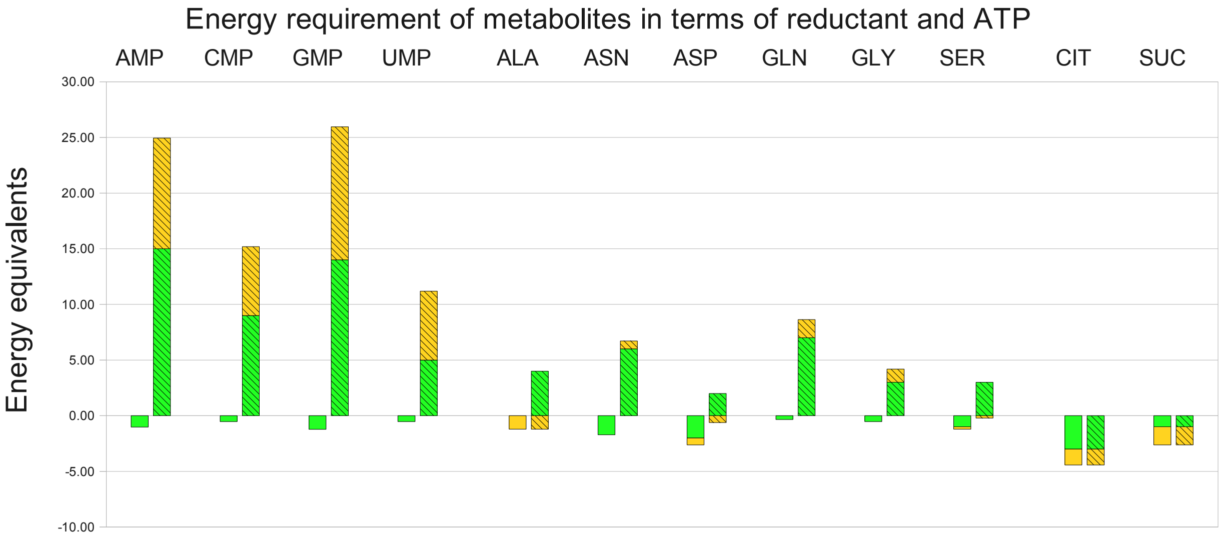

Figure 3: Theoretically calculated energy and reductant balance to produce important metabolites from starch. The bars indicate the amount of ATP (yellow) and NADPH (green) equivalents, which are minimally required to produce the respective metabolite (see Methods). Negative numbers depict a surplus. Numbers are shown for ammonium (clear) and nitrate (hatched) as the only nitrogen source.

amino acids than cells grown on mixed ammonium and nitrate [16].

These findings give rise to the question how much carbon contained in transient starch can *in principle* be used to produce biomass and how much needs to be respired in order to provide the necessary energy. To answer this question we calculated flux distributions producing a given amount of biomass under two objectives. The first objective demanded that the resulting flux distribution minimises the overall flux through all reactions (flux minimisation – FM) assuming equal weights for all reactions, and the second minimised the carbon which is exported (as CO_2 or other organic compounds). The second objective gives the theoretical optimum of carbon conversion from starch to biomass.

Again, the results depend strongly on the nitrogen source. We found that, growing on ammonium, 92.2% of starch carbon can theoretically be converted into biomass (FM yielded the slightly lower value of 92.0%). On nitrate, however, a lower fraction of 79.2% (78.2% under FM) can theoretically be converted. In all cases, the remaining fraction of starch-derived carbon was respired and exported as CO_2. Similarly as for the single compounds, the reduced fractions on nitrate can readily be explained by the fact that the reduction of nitrate requires a considerable amount of reductant. It is interesting that both objectives give very similar results. The small discrepancies are explained by the fact that FM favours shorter pathways, which sometimes are less energy efficient. It should be stressed that these figures overestimate the carbon yield, because the calculation neglects any energy or reductant requirement for maintenance purposes. Still, it is remarkable that our results reflect experimentally observed differences in growth rates on nitrate and ammonia [17] and comparison to real growth rates during the night (see Discussion) may be helpful to understand the quantity and nature of

maintenance metabolism.

Nitrogen metabolism in the two tissue model cont'd The scan presented in Fig. 4 visualises the gradual change of fluxes with changing nitrogen source, presented in Figure 2 of the main text. As explained, the amount of required starch (dash-dotted dark blue) drops with increasing availability of ammonium. This directly reflects the higher costs for nitrate assimilation. However, with more available ammonium, the activity of the mitochondrial respiratory chain and, concomitantly, the mitochondrial ATP synthase (dotted light blue) are increased. This observation can be explained by considering the changes in redox balance: More nitrogen available as ammonium results in a reduced reductant requirement to reduce nitrate. Therefore, more of the reductant produced in the TCA cycle (dotted green) can be used to generate ATP. The plot simultaneously shows the change of proton (dashed orange line) and carbonate (dashed purple) export to the soil.

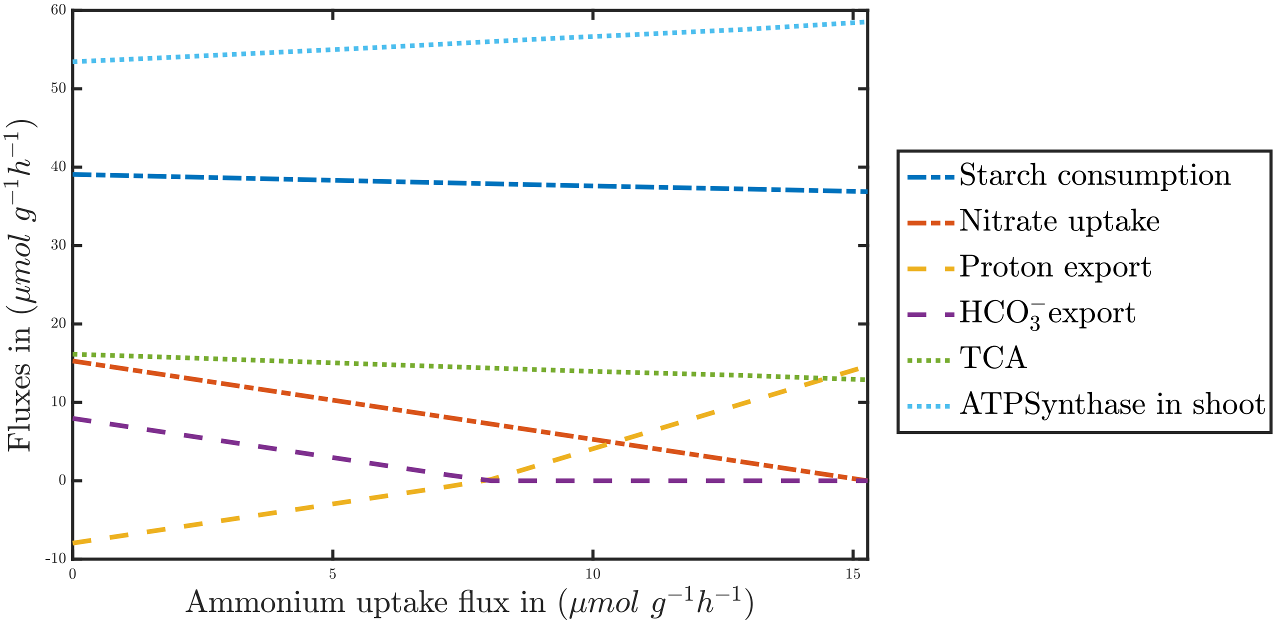

Figure 4: Responses of metabolic fluxes for increasing availability of ammonium. Maximal ammonium uptake (x-axis) was systematically varied and the response of selected fluxes depicted (y-axis). The fluxes were calculated by fixing the biomass production to 0.1 g/gDW per day and minimising the total quadratic flux through the network.

2 Materials and Methods Supplement

2.1 Metabolic Model Construction

The model construction procedure is summarised in Figure 5. Depicted are key steps in model development and the required data source to perform each step.

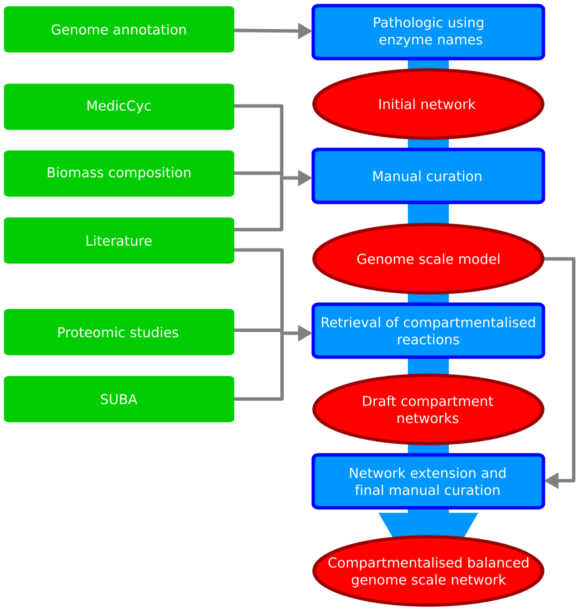

Figure 5: Layout of the model generation process. Data sources are marked in green, model stages in red and process steps blue. During manual curation producibility of all measured compounds was ensured and all reactions where charge balanced.

Initial Network Generation. The model is based on version 3.5v5 of the *Medicago truncatula* genome annotation [1]. The initial construction was performed using PathoLogic enzyme name matching from the PathwayTools program [4] and makes use of the MedicCyc (v1.0) database [2], which is based on the BioCyc [3] suite of databases. Information from MedicCyc (v1.0) was incoporated based on homology analysis between gene information in MedicCyc and the 3.5v5 genome annotation. This resulted in a list of enzyme-catalysed reactions converting mainly small molecules. However, the MetaCyc database also includes a number of reactions which contain macromolecules. In general, metabolic network models are limited to reactions acting on small molecules. Macromolecules, such as proteins or glucans, are not specifically included because of the enormous number of theoretically possible polymers. However, some macromolecules play important roles in metabolism and require special treatment. Among these are in particular small carrier proteins such as ferredoxins (for charge transfer reactions) or acetyl carrier proteins (for fatty acid biosynthesis). As essential cofactors, these molecules have to be explicitly included in the reaction stoichiometries but their synthesis pathways are outside the scope of a metabolic network model. To verify mass balance of reactions involving such molecules, we exploit that these macromolecules in their various modified forms form conserved moieties, i. e. that their core structures are not changed by any reaction in the network. For reactions involved in synthesis or degradation of polymeric sugars, such as starch, we replaced the compounds representing the polymers of a non-specified degree of polymerisation (such as starch(n)) by the monomers in such a way that the overall reaction is correctly balanced. To this extent, a xylan polymer for example is assumed to consist

of dehydrated xylose subunits (C5H8O4).

Some reactions added to the model by PathwayTools had poor evidence. The name matching algorithm adds reactions if the assigned enzyme names in MetaCyc match those in the genome annotation, which leads to problems with unspecific annotations such as "alcohol dehydrogenase" or "aldehyde dehydrogenase". Such descriptors resulted in the addition of many reactions that were disconnected from the remaining network.

We therefore removed reactions from the model which fulfilled all of the following conditions: 1) a contained metabolite is only present in this reaction, 2) the reaction cannot carry any flux under the condition that all nutrients can be imported and all metabolites can be exported, and 3) there exists no evidence for the presence of the reaction beyond a match of unspecific reaction names.

Gap filling. Gaps in the initial network reconstruction were identified by verifying that all experimentally observed metabolites were actually producible from the nutrients. For this, we collected a list of experimentally verified metabolites from various studies [18, 19, 20, 21, 22, 23]. We then computed for each of these metabolites whether a stationary flux distribution exists (see below) such that this metabolite is produced while only the nutrients are consumed. When this check failed, the MetaCyc database was searched for plausible candidate reactions which needed to be added in order to allow for a production of the respective metabolite. The identified reactions were manually added to the network.

Atomic and Charge balancing. We intended to create a model which can simulate both photosynthetic and heterotrophic growth. Because proton gradients are essential in both regimes, we needed to ensure all reactions are charge balanced. Using MetaCyc charge information (all compounds protonated to a pH of 7.3) we checked all reactions for their charge balance. Apparently, several MetaCyc reactions have been curated to achieve mass balance by adding protons. This is in particular true for reactions in the phospholipid desaturation pathway, but also in reactions representing dehydrogenase or mixed function oxygenase activities. Where possible, we curated these reactions by assuming common desaturase, dehydrogenase or mixed function oxidase activities. In general, when protons had been added for mass balance reasons they were replaced by NAD(P)H + H^+/NAD(P)$^+$ pairs to achieve charge balance. This replacement was done by applying the general rules which are laid out in Table I.

Atomic balance was checked using the method detailed in Gevorgyan et al.[24] and only light was shown as unconserved. This is to be expected, as light does only transfer energy into the system and does not contribute towards atomic balance.

2.2 Model Compartmentalisation

In order to realistically describe the metabolism of a plant cell, it is important to develop a fully compartmentalised model. Each compartment fulfils specialised tasks while key processes, such as ATP generation, are only possible due to gradients established over inter-compartmental membranes. Key challenges when building a compartmentalised model are a) to realistically assign compartments to each reaction, b) to define transport reactions over intracellular membranes and c) to ensure that the resulting model is not able to generate and maintain gradients which are thermodynamically infeasible.

Assigning compartments to reactions To overcome the first challenge, we applied the following approach: We first scanned all available protein sequences for annotated genes against the *Arabidopsis thaliana* genome on TAIR [25] and extracted information about localisation from the SUBA database [7]. We assigned compartments to only those reactions for which experimental evidence, such as GFP-localisation, MS identification, TAIR data, and swissprot data, existed. Because computational predictions are known to result in a large number of false positives, they were not included during this step. In addition, two recent proteomic studies for root plastids [5] and mitochondria [6] in *M. truncatula* were used to assign compartments to reactions. For this, the provided gene identifiers, which were based on different versions of the Medicago truncatula Gene Index [26], were translated to gene identifiers of version 3.5v5 of the *M. truncatula* genome annotation using BLAST. All these sources of information were integrated and if evidence for the presence of a protein in a specific compartment was found in at least one source it was added to this compartment. This initial process resulted in a draft compartmentalised network with still a large number of reactions not yet assigned to compartments. In a next step, we used an established network modelling approach [8] to ensure that each compartment contains a self-consistent metabolic network: The network extension developed by Christian et al. identifies a minimal set of reactions from a reference network which need to be added to a draft network

	Replacement example	Assumed enzymatic activity
Original reaction<br>Corrected reaction	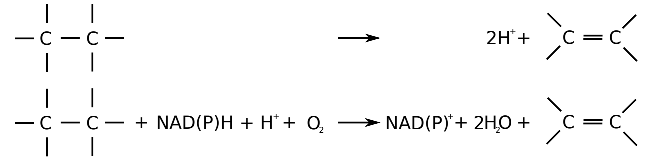	desaturase
Original reaction<br>Corrected reaction	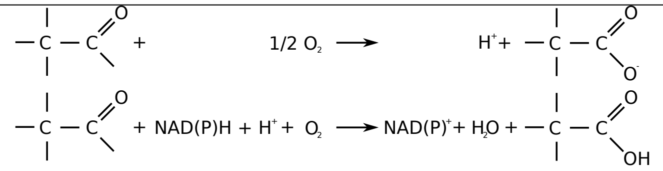	aldehyde dehydrogenase

Table I: Examples for the replacement of reactions that are not charge balanced by plausible enzymatic activities, which ensure a correct charge balance.

in order to ensure that a number of 'seed' metabolites can be metabolised into a number of 'targets'. While for a whole organism, the seed can directly be inferred from defined growth media, and the targets can be obtained by metabolomic data, their definition is not straight-forward for single compartments. We obtained seed and target sets for each compartment by an extensive literature research for metabolites which are known to be imported into the compartment or produced by the compartment. This step was performed for all compartments except the cytosol and the vacuole. This procedure is difficult for the cytosol, because it is in direct contact to all other compartments. Similarly, the vacuole is a main location for degradation and storage, making a meaningful definition of seeds and targets difficult. For the vacuole we therefore used the gene localised reactions for the initial compartment. We manually assigned additional reactions only if they were missing steps in degradation pathways for which a majority of reactions was already present in the vacuole. Whereas in network extension for whole organism networks, usually a network containing all reactions stored in a biochemical database is used as a reference, we here use all reactions of the uncompartmentalised network as reference, because our goal is to assign each reaction a particular compartment. The lists of seed and target metabolites used for this process and the resulting reactions added to each compartment can be found in Supplementary Table S1. All reactions which remained without assigned compartment after applying network extension to all compartments were subsequently assigned to the cytosol, with the exception of the sterol metabolism which was assigned to the ER.

Transporters Describing transport processes of metabolites over membranes is a challenge in all compartmentalised metabolic networks. The main difficulty is that exact knowledge about transported metabolites is still missing. Further, the existence of several transporters has to be assumed without experimental evidence due to localisation of biosynthetic enzymes. Recently, Linka et al. reviewed many characterised transport systems for plants [13]. However, even this comprehensive review must necessarily miss transporters for which no molecular characterisation exists yet, but which are essential for production of different metabolites. We added transporters between compartments to ensure that all metabolites which were producible in the uncompartmentalised network were still producible in the compartmentalised network. For this, we used the information given in Linka et al. [13] complemented by several other sources [10, 11, 12], and otherwise included transporters which seemed biologically most plausible. In the presented model, we assume that most transporters do not carry a net charge. This is true in many instances, but more detailed information on the charges carried by transporters would further improve the model description and provide further insights [27]. A full list of added transporters is given in Supplementary Table S4.

Maintaining thermodynamic integrity Another challenge arises with protonation states of different compounds. This is particularly important for the mitochondrial compartment which harbours the electron

transfer chain for ATP synthesis. Because there are plenty of transporters over the mitochondrial membrane, we had to ensure in the model description that these transporters do not allow a net-charge exchange over the membrane, because otherwise proton gradients might be generated within the model by a simple shuttling of metabolites. This would result in the generation of ATP uncoupled from energy provided by exergonic reactions, thus rendering the model thermodynamically infeasible and predictions regarding ATP generating fluxes meaningless. All transporters over the mitochondrial and plastidial membranes were therefore balanced for charges by adding protons in a way that a net charge of zero is translocated. The only exception to this were reactions from the electron transfer chain for which proton translocation is known. Similarly, import and export of metabolites over the cellular membrane is critical for charge balancing the system. We include uptake and export of charged particles by importers and exporters. Additionally, we introduce a proton and bicarbonate exchanger. In our simulations, we applied the constraint (see below) that the net charge exchange is zero. This resulted in the model behaviour that protons and bicarbonate were used to balance excess uptake or export of charges. The biomass reaction itself was charge-balanced by adding protons to ensure that the net charge of the biomass is zero.

2.3 Generation of a two tissue shoot-root Network

To more realistically describe processes taking place in root cells, we have constructed a tissue-specific sub-network. We used the presence calls provided in Benedito et al [28] to determine core reactions sets for shoots and roots. We assume a gene to be present if it is determined as present in a majority of experiments. Shoot core genes are the combination of all genes present in stems and leaves. Root core genes are those genes determined as present in the root. The core reaction sets used for the model generation were determined by matching the determined genes using the gene-protein-reaction association rules in the model, and determining which reactions could be activated by the present genes. The model was then duplicated as a root and a shoot model and exchangers between the two tissues were added similar to the concept detailed in A full list of tissue exchanges is provided in Supplemental Table S3. All uptakes from the external medium except for CO_2, light and starch were removed from the leave model, and glucose, starch, light and sucrose exchangers were removed from the root model. This combined model was made consistent, i.e. all reactions which could not carry flux were removed using FASTCC [29]. The consistent model was then subjected to the FASTCORE algorithm [29]. FASTCORE extracts a small consistent network from the original network, in which all reactions from a given core set can be activated. We used those reactions as core for which the models gene-protein-reaction relationships returned true, if the core genes for the respective tissue were set as active. In addition, some reactions were turned off to generate the individual models. This step was repeated four times with slightly modified models to obtain four individual conditions:

1. Growth using ammonium as sole nitrogen source during the day.
2. Growth using ammonium as sole nitrogen source during the night.
3. Growth using nitrate as sole nitrogen source during the day.
4. Growth using nitrate as sole nitrogen source during the night.

For day conditions, the biomass reaction without starch in the leaves, and starch import were turned off, while night conditions had the starch containing biomass and light import turned off. For growth on nitrate, the ammonium uptake was shut down and for growth on ammonium, the nitrate exchanger was set to 0, respectively. Day conditions implied production of a biomass containing starch along with availability of light, while night conditions only allowed starch as a carbon and energy source while producing a biomass without starch. Finally, all reactions present in at least one of these four models were combined to yield the two tissue model. Performing only one FASTCORE calculation could have led to disconnected subnetworks, where e.g. light could be used to fix carbon but the carbon might not be linked to the remaining network. We employed multiple constraints for the analysis of the model. An important factor, atp maintennce, was estimated based on data from DeVries et al.[30] for *Trifolium repens*, white clover, the closest relative for which data is available. This plant needs 15 mg glucose per gram dry weight per day for maintenance [30]. With a regenerating ability of about 32mol ATP per mol glucose, this translates to an hourly maintenance cost of about 111.1 µmol/(g h), a number which has to be distributed between the two tissues. With an approximate 1:2 ratio (root:shoot) the atp maintenance for shoot was set to 74 µmol/(g h) and the root maintenance set to 37 µmol/(g h) Similarily the biomass production combines two thirds of a shoot biomass and one third of a root biomass to one μmol of biomass, with a weight of 1 g. We assumed a normal growth rate of 0.1 g/(g d) based on data for *M. sativa* [31] for simulations where a fixed biomass production was required. The maximal starch consumption in leaves was set to 46.32 µmol/(g h), based on the starch storage in our biomass experiments during the day. The maximal rate of photon uptake was set to 1000 µmol/(g h).

2.4 Building a symbiotic system

To be able to compare fluxes in the rhizobium with fluxes in the plant, we had to adjust all fluxes to use a common unit, which we based on plant dry weight. To achieve this we used the following data: The ratio between nodule dry weight and plant dry weight is in the range between 0.01 g/g [32] and 0.035 g/g [33]. In addition, there are roughly $8 \cdot 10^{10}$ bacteroids per gram nodule [34] and a bacteroid weights approximately $3 \cdot 10^{-12}$ g [35]. Thus the total amount of bacteroids per gram of plant dry weight is in between 2.40 mg/g and 8.40 mg/g. Table II lists the original and adapted ranges of fluxes. We assumed a symbiont maintenance

Flux	Experimental value $nmol\ mg^{-1}\ min^{-1}$ (based on bacteroid weight)	Model constraint $\mu mol\ g^{-1}\ h^{-1}$ (based on plant weight)	Source
Succinate uptake	22.1	0 - 9.4248	(1)
Malate uptake	18.7	0 - 11.1384	(1)
Total dicarboxylate uptake	-	0-11.1384	
Amino acid uptake	75.8	0 - 37.99*	(1)
Oxygen uptake	14.1 - 39.2	16.8*2	(2)

Table II: Flux constraints for metabolite exchange between rhizobium and plant. For conversion between bacteroid and plant weight a ratio of 8.4 mg/g was assumed.
*: This constraint was used to limit the total amino acid exchange (uptake and export).
*2: The listed oxygen uptake is in the upper range of possible uptake rates. When alternate values are used this is explicitly mentioned.
(1):[37] , (2):[35]

energy of 7.6 mmol/(g h) based on symbiont weight which translates to 63.47 µmol/(g h) based on plant weight. This is the maintenance energy commonly used in *E.coli* [36]. After combining the models, we noticed, that our biomass formulation has a very large asparagine fraction. This is likely due to the fact, that we fed our plants with excessive amounts of nitrogen to avoid any nodulation. Therefore, we reduced the amount of free asparagine in the plant biomass formulation to 5% of the original amount. Without this adjustment, the maximal growth rate with maintenance energy is at about 0.04 g/(g d), which is about 53% lower than with the adjusted amount. In addition, the rhizobium spotted a citrate lyase reaction, which by acting in reverse allowed the production of some ATP. Since this protein commonly catalyses the forward reaction, we set its lower bound to zero. For our simulations, the symbiont was assumed to have finally differentiated and therefore stopped growth, i.e. not consume the nitrogen it fixes, de facto becoming an additional compartment of the plant.

2.5 Network analysis

Genome-scale networks are most conveniently described by the stoichiometry matrix $\mathbf{N}$, in which columns represent biochemical reactions and rows correspond to metabolites, and the coefficients define whether a metabolite is consumed (negative) or produced (positive) by a particular reaction. The dynamic behaviour of the metabolic network is governed through the stoichiometry matrix by

$$\frac{d\mathbf{S}}{dt} = \mathbf{N} \cdot \mathbf{v}(\mathbf{S}, \mathbf{p}), \tag{1}$$

where $\mathbf{S}$ is the vector of metabolite concentrations, $\mathbf{v}$ is the vector containing all reaction rates and $\mathbf{p}$ is a vector with all kinetic parameters. Assuming that the system is in stationary state, the dynamic equation is

reduced to the stoichiometric equation

$$\mathbf{N} \cdot \mathbf{v} = 0, \tag{2}$$

which allows to derive statements about the flux distribution $\mathbf{v}$ from the stoichiometry matrix alone. This system is usually under-determined, meaning that many flux distributions fulfil the equation. To restrict the solution space, further constraints are defined. These reflect thermodynamic constraints restricting the direction of some reactions, as well as experimentally observed constraints, such as maximal uptake rates and measured growth rates combined with biomass composition. Such limitations are in the most general form described by inequalities

$$l_j \leq v_j \leq u_j, \tag{3}$$

where l_j and u_j are lower and upper bounds restricting the flux through a reaction or transporter j. In our case, an important constraint also reflects the fact that the net charge accumulation must be zero. This constraint is written as

$$\sum_i q_i \cdot v_i = 0, \tag{4}$$

where q_i is the net charge translocated over the cellular membrane by transporter i. Network analysis was performed using ScrumPy [38] (`mudshark.brookes.ac.uk/ScrumPy`) and the COBRA toolbox [39] with CPLEX as solver.

Producibility of metabolites To verify whether a particular metabolite X is producible, a reaction $v_k : X \rightarrow \emptyset$ is added to the network. Then, it is tested whether a solution vector $\mathbf{v}$ exists, such that the stationarity condition 2 and the constraints 3 and 4 are fulfilled, for which $v_k > 0$. If the system is solvable, there exists a stationary flux distribution which consumes only nutrients and produces (at least) metabolite X.

Energy and redox requirements To find a theoretical minimum of energy and reductant requirements to produce a certain metabolite k, we introduce as above a reaction $v_k : X \rightarrow \emptyset$ and two reactions consuming (positive flux) or producing (negative flux) energy and redox equivalents (the ATPase, ATP + H_2O + H^+ $\rightarrow$ ADP + Pi, and the NADPH dehydrogenase, NADPH + H^+ + 0.5 O_2 $\rightarrow$ NADP $^+$ + H_2O). The optimisation problem is to find a flux distribution which produces one unit flux through the reaction consuming metabolite k, while minimising the fluxes through the ATPase and NADPH dehydrogenase. For this, a three step optimisation is performed. First both ATPase and dehydrogenase remain unconstrained with the optimisation being a minimisation of starch consumption. The next step is to fix starch consumption and minimise the flux through the dehydrogenase reaction. The final step is to also fix the dehydrogenase flux and minimise the flux through the ATPase reaction.

Flux Balance Analysis The identification of special solutions, which optimise some plausible objective function, is subject of Flux Balance Analysis (FBA, [40]). The choice of the objective function is an unsolved problem. A common assumption is that fluxes are arranged such that biomass yield is maximised [41]. However, this assumption is experimentally supported only for *E. coli* [42, 43, 44] and the objective is certainly debatable for eukaryotic and multicellular organisms. Different tissues of a complex organism and likewise different compartments in a eukaryotic cell will have different functions and therefore follow different objectives. Unless otherwise stated, we perform our calculations by solving the optimisation problem to find a flux distribution, that minimises the quadratic fluxes. Due to the properties of the quadratic problem, this solution is necessarily unique thus avoiding problems with alternate optima. While this objective may not reflect the true objective, it reflects a plausible assumption and allows to investigate how the most economic flux distribution changes upon external perturbations. To calculate theoretically most efficient conversions during night, we introduce 'carbon optimisation'. Here, a flux distribution is determined which minimises export of carbon (e. g. as CO_2) in a linear fashion and thus ensures a maximal ratio of carbon from starch being incorporated into the biomass. In all simulations where flux minimisation was desired, the glucose exporter was set to zero, as otherwise, glucose is exported when starch is degraded. The unrealistic solution is obtained because if maltose is split into glucose and glucose phosphate and unphosphorylated glucose is exported, a slightly lower overall flux is obtained than for the more realistic solution in which the extra ATP is produced which is required to phosphorylate the second glucose.

Performance scans of metabolism Some demands on the metabolic network are unknown. It is, for example, unknown how much energy is required for maintenance or how many reductants are required to maintain the redox balance. By fixing fluxes representing these requirements to a certain value and systematically varying its quantity we can investigate how the system responds to external challenges. For this, an additional constraint, $v_i = \alpha$, is introduced and the equation system is solved for a certain range of values of α. To determine varying energy and redox demands, we introduce two additional reactions, an ATP hydrolysis (ATPase: ATP + H_2O + H^+ $\rightarrow$ ADP + Pi) and a dehydrogenase-like reaction (DEHOG: NADPH + H^+ + 0.5 O_2 $\rightarrow$ NADP $^+$ + H_2O). When not mentioned otherwise, these reactions are The values of these fluxes are fixed and the system is solved for a range of values.

Hydroponic culture of *M. truncatula* Seeds were scarified for 8 minutes using 95/97% H_2SO_4. After washing they were incubated in a hydrochloric solution (Millipore tablets 2WCL01F50) for 30 minutes and finally washed with a large surplus of sterilized dH_2O. The seedlings were transferred to agar plates (0.8% (w/v)) and left in the dark at 25 °C for 48 hours. Plants were grown hydroponically for eight weeks at 21 °C and 16/8h Light/dark cycle. The nutrient solution was prepared according to the *Medicago truncatula*

Handbook [45] with a final ammonium nitrate concentration of $8\,\mathrm{mmol\,L^{-1}}$ and no potassium nitrate. The tubs were aerated with air stones. 8 week old plants were harvested in 2 intervals. One harvest at the end of the light period, the other at the end of the dark period. After harvest, plants were submerged in liquid nitrogen and freeze dried till further use.

Extraction of soluble metabolites Soluble metabolites were extracted from lyophilized tissue using methanol/chloroform according to the protocol of Lisec et al.[46]. Tissue samples (leaves, root, stem) were transferred to a mortar and ground to a fine powder under liquid nitrogen. A known mass (25 mg) was weighed into a pre-cooled 15 mL Falcon tube and 7 mL of 100% Methanol (pre cooled at −20 °C) was added and vortexed for 10 s. 300 µL of ribitol ($0.2\,\mathrm{mg\,mL^{-1}}$) was added as internal standard at this point. Tubes were shaken for 10 min at 70 °C. 730 µL were transferred to 2 mL eppendorf tubes and the tubes were centrifuged at 11,000 g for 10 min to pellet insoluble material. 375 µL chloroform and 750 µL water (precooled to -20 and 4 °C respectively) were added. The tubes were briefly vortexed and centrifuged for 15 min at 2,200 g to allow the non-polar and polar phases to separate. For GC-MS analysis aliquots of the polar phase were taken and dried down. Lipids were extracted using Hexane/Isopropanol extraction according to Hara et al.[47] and quantified by weight.

Protein extraction, precipitation and hydrolysis Protein extraction, precipitation and hydrolysis was performed according to Williams et al.[48] using a Urea/Thiourea extraction. Analysis was performed by GC-MS. twice with 4 mL of ice-cold acetone and again centrifuged as above. The pellets were allowed to stand in a flow hood to evaporate any acetone left. Protein weights were taken after drying overnight. The protein pellets were re-suspended in 2 mL of 6M HCl and incubated in a heat block at 100 °C for 24 hours for hydrolysis. Samples were cooled to room temperature and an aliquot (20 µL to 50 µL) of the hydrolysates (containing amino acids) were transferred to eppendorf tubes and dried in a vacuum dryer overnight to remove HCl. The dried samples were subjected to GC-MS analysis.

Nucleic acids RNA and DNA were extracted using TRIzol/DNAse and Chloroform/isoamylalcohol extraction methods respectively (adapted from [49]). Quantification was performed photospectrometrically using a nanodrop (ND-1000 Spectrophotometer; NanoDrop Technologies Inc.; Wilmington, DE, USA).

Starch Starch was extracted according to an established protocol [48]. The insoluble pellet obtained after the methanol chloroform extract was used as starting point. Each sample was gelatinized by autoclaving the residue in 3 mL of 25 mM sodium acetate for 3 h at 121 °C, 1.04 bars pressure followed by enzymatic digestion with 20 U α-amylase (Sigma-Aldrich) and 5 U amyloglucosidase (Sigma-Aldrich) for 16 h at 37 °C.

The samples were centrifuged and the supernatant containing the glucose monomers was collected, freeze dried and stored at −80 °C until further analysis. Starch amounts were determined with an enzymatic assay.

Gas chromatography-mass spectrometry GC-MS analysis was done as in Masakapalli et al.[16] and Williams et al.[48] using an Agilent 79890 GC coupled to an Agilent 5975 quadrupole MS detector, electron impact ionisation (70 eV) equipped with an Agilent HP 5-ms column (30 mm, 0.25 mm inner diameter) at the facility in the Department of Plant Sciences, University of Oxford, UK.
The protein hydrolysate extracts were derivatised by TBDMS [50] whereas the soluble extracts were derivatised by MeOX TMS [46]. To obtain the TBDMS derivatives, the dried samples were first dissolved in 25 µL of pyridine and incubated at 37 °C, shaking at 900 rpm for 30 min. Then 35 µL of MtBSTFA + 1% t-BDMCS (N-methyl-N-(t-butyldimethylsilyl) trifluoroacetamide + 1% t-butyl-dimethylchlorosilane, Regis Technologies Inc) was added and the mixture was incubated at 60 °C, shaking at 900 rpm for 30 min. To obtain the MeOX TMS derivatives, 40 µL of 20 mg mL^{-1} methoxyamine hydrochloride (Sigma) in pyridine was added to the sample and incubated at 37 °C, shaking at 900 rpm for 2 h. Then 70 µL MSTFA (N-methyl-N-(trimethylsilyl)trifluroacetamide (HiChrom)) was added and the mixture was incubated at 37 °C for 30 min at 900 rpm. The derivatised samples were transferred to 8 mm glass vials (Chromacol) and sealed with a septum cap. The samples were run through GC-MS. The mass spectra of all the samples were acquired for m/z 146-600 by scanning (at 4.38 or 3.19 scans s^{-1}). A solvent delay of 10 min was set so that the mass spectrometer was turned on after elution of the bulk of the solvent from the column.

References

[1] Young, N. D. *et al.* The medicago genome provides insight into the evolution of rhizobial symbioses. *Nat.* **480**, 520–524 (2011).

[2] Urbanczyk-Wochniak, E. & Sumner, L. W. Mediccyc: a biochemical pathway database for medicago truncatula. *Bioinforma.* **23**, 1418–1423 (2007).

[3] Caspi, R. *et al.* The MetaCyc database of metabolic pathways and enzymes and the BioCyc collection of pathway/genome databases. *Nucleic Acids Res* **38**, D473–D479 (2010). DOI 10.1093/nar/gkp875.

[4] Karp, P. D. *et al.* Pathway tools version 13.0: integrated software for pathway/genome informatics and systems biology. *Briefings Bioinforma.* **11**, 40–79 (2010). DOI 10.1093/bib/bbp043.

[5] Daher, Z. *et al.* Proteomic analysis of medicago truncatula root plastids. *Proteomics* **10**, 2123–2137 (2010). DOI 10.1002/pmic.200900345.

[6] Dubinin, J., Braun, H.-P., Schmitz, U. & Colditz, F. The mitochondrial proteome of the model legume medicago truncatula. *Biochimica et Biophys. Acta (BBA) - Proteins & Proteomics* **1814**, 1658–1668 (2011).

[7] Heazlewood, J. L., Verboom, R. E., Tonti-Filippini, J., Small, I. & Millar, A. H. Suba: the arabidopsis subcellular database. *Nucleic Acids Res.* **35**, D213–D218 (2007). DOI 10.1093/nar/gkl863.

[8] Christian, N., May, P., Kempa, S., Handorf, T. & Ebenhöh, O. An integrative approach towards completing genome-scale metabolic networks. *Mol Biosyst* **5**, 1889–1903 (2009). DOI 10.1039/B915913b.

[9] Benveniste, P. Biosynthesis and accumulation of sterols. *Annu. Rev Plant Biol* **55**, 429–457 (2004). DOI 10.1146/annurev.arplant.55.031903.141616.

[10] Babujee, L. *et al.* The proteome map of spinach leaf peroxisomes indicates partial compartmentalization of phylloquinone (vitamin k1) biosynthesis in plant peroxisomes. *J. Exp. Bot.* **61**, 1441–1453 (2010).

[11] Helliwell, C. A. *et al.* A plastid envelope location of arabidopsis ent-kaurene oxidase links the plastid and endoplasmic reticulum steps of the gibberellin biosynthesis pathway. *Plant J* **28**, 201–208 (2001).

[12] León, J. & Sánchez-Serrano, J. J. Molecular biology of jasmonic acid biosynthesis in plants. *Plant Physiol. Biochem.* **37**, 373–380 (1999).

[13] Linka, N. & Weber, A. P. Intracellular metabolite transporters in plants. *Mol. Plant* **3**, 21–53 (2010). DOI 10.1093/mp/ssp108.

[14] Benyamini, T., Folger, O., Ruppin, E. & Shlomi, T. Flux balance analysis accounting for metabolite dilution. *Genome Biol* **11**, R43 (2010). DOI 10.1186/gb-2010-11-4-r43.

[15] Kruse, K. & Ebenhöh, O. Comparing flux balance analysis to network expansion: producibility, sustainability and the scope of compounds. *Genome Inf.* **20**, 91–101 (2008).

[16] Masakapalli, S. K., Kruger, N. J. & Ratcliffe, R. G. The metabolic flux phenotype of heterotrophic arabidopsis cells reveals a complex response to changes in nitrogen supply. *Plant J* **74**, 569–582 (2013). DOI 10.1111/tpj.12142.

[17] Brix, H., Dyhr-Jensen, K. & Lorenzen, B. Root-zone acidity and nitrogen source affects typha latifolia l. growth and uptake kinetics of ammonium and nitrate. *J Exp Bot* **53**, 2441–2450 (2002).

[18] Barsch, A., Carvalho, H. G., Cullimore, J. V. & Niehaus, K. Gc-ms based metabolite profiling implies three interdependent ways of ammonium assimilation in medicago truncatula root nodules. *J. Biotechnol.* **127**, 79–83 (2006).

[19] Broeckling, C. D. *et al.* Metabolic profiling of medicago truncatula cell cultures reveals the effects of biotic and abiotic elicitors on metabolism. *J Exp Bot* **56**, 323–336 (2005). DOI 10.1093/jxb/eri058.

[20] Farag, M. A., Huhman, D. V., Lei, Z. & Sumner, L. W. Metabolic profiling and systematic identification of flavonoids and isoflavonoids in roots and cell suspension cultures of medicago truncatula using hplc-uv-esi-ms and gc-ms. *Phytochem.* **68**, 342 – 354 (2007). DOI DOI: 10.1016/j.phytochem.2006.10.023.

[21] Huhman, D. V. & Sumner, L. W. Metabolic profiling of saponins in medicago sativa and medicago truncatula using hplc coupled to an electrospray ion-trap mass spectrometer. *Phytochem.* **59**, 347 – 360 (2002). DOI DOI: 10.1016/S0031-9422(01)00432-0.

[22] Kowalska, I. *et al.* Flavonoids from barrel medic (medicago truncatula) aerial parts. *J. Agric. Food Chem.* **55**, 2645–2652 (2007). DOI 10.1021/jf063635b. `http://pubs.acs.org/doi/pdf/10.1021/jf063635b`.

[23] Pollier, J., Morreel, K., Geelen, D. & Goossens, A. Metabolite profiling of triterpene saponins in medicago truncatula hairy roots by liquid chromatography fourier transform ion cyclotron resonance mass spectrometry. *J. Nat. Prod.* **74**, 1462–1476 (2011).

[24] Gevorgyan, A., Poolman, M. G. & Fell, D. A. Detection of stoichiometric inconsistencies in biomolecular models. *Bioinforma.* **24**, 2245–2251 (2008). DOI 10.1093/bioinformatics/btn425.

[25] Lamesch, P. *et al.* The arabidopsis information resource (tair): improved gene annotation and new tools. *Nucleic Acids Res.* **40**, D1202–D1210 (2012). DOI 10.1093/nar/gkr1090.

[26] Quackenbush, J. *et al.* The tigr gene indices: analysis of gene transcript sequences in highly sampled eukaryotic species. *Nucleic Acids Res* **29**, 159–164 (2001).

[27] Cheung, C. Y. M. *et al.* A method for accounting for maintenance costs in flux balance analysis improves the prediction of plant cell metabolic phenotypes under stress conditions. *Plant J* (2013). DOI 10.1111/tpj.12252.

[28] Benedito, V. A. *et al.* A gene expression atlas of the model legume *Medicago truncatula*. *Plant J* **55**, 504–513 (2008). DOI 10.1111/j.1365-313X.2008.03519.x.

[29] Vlassis, N., Pacheco, M. P. & Sauter, T. Fast reconstruction of compact context-specific metabolic network models. *PLoS Comput. Biol* **10**, e1003424 (2014). DOI 10.1371/journal.pcbi.1003424.

[30] De Vries, F. W. T. P. The cost of maintenance processes in plant cells. *Annals Bot.* **39**, 77–92 (1975).

[31] Lötscher, M., Klumpp, K. & Schnyder, H. Growth and maintenance respiration for individual plants in hierarchically structured canopies of medicago sativa and helianthus annuus: the contribution of current and old assimilates. *New Phytol.* **164**, 305–316 (2004).

[32] Vance, C. P., Heichel, G. H., Barnes, D. K., Bryan, J. W. & Johnson, L. E. Nitrogen fixation, nodule development, and vegetative regrowth of alfalfa (*Medicago sativa* l.) following harvest. *Plant Physiol* **64**, 1–8 (1979).

[33] Aydi, S., Drevon, J.-J. & Abdelly, C. Effect of salinity on root-nodule conductance to the oxygen diffusion in the *Medicago truncatula-Sinorhizobium meliloti* symbiosis. *Plant Physiol Biochem.* **42**, 833–840 (2004). DOI 10.1016/j.plaphy.2004.10.003.

[34] Sutton, W. D., Jepsen, N. M. & Shaw, B. D. Changes in the number, viability, and amino-acid-incorporating activity of *Rhizobium* bacteroids during lupin nodule development. *Plant Physiol* **59**, 741–744 (1977).

[35] Bergersen, F. Physiological and biochemical aspects of nitrogen fixation by bacteroids in soybean nodule cells. *Soil Biol Biochem.* **29**, 875–880 (1997).

[36] Orth, J. D. *et al.* A comprehensive genome-scale reconstruction of escherichia coli metabolism–2011. *Mol Syst Biol* **7**, 535 (2011). DOI 10.1038/msb.2011.65.

[37] McRae, D. G., Miller, R. W., Berndt, W. B. & Joy, K. Transport of c(4)-dicarboxylates and amino acids by *Rhizobium meliloti* bacteroids. *Mol Plant-Microbe Interact* **2**, 273–278 (1989). DOI 10.1094/MPMI-2-273.

[38] Poolman, M. G. Scrumpy: metabolic modelling with python. *Syst Biol (Stevenage)* **153**, 375–378 (2006).

[39] Schellenberger, J. *et al.* Quantitative prediction of cellular metabolism with constraint-based models: the cobra toolbox v2.0. *Nat Protoc* **6**, 1290–1307 (2011). DOI 10.1038/nprot.2011.308.

[40] Kauffman, K. J., Prakash, P. & Edwards, J. S. Advances in flux balance analysis. *Curr Opin Biotechnol* **14**, 491–496 (2003).

[41] Feist, A. M. & Palsson, B. O. The biomass objective function. *Curr Opin Microbiol* **13**, 344–349 (2010). DOI 10.1016/j.mib.2010.03.003.

[42] Edwards, J. S. & Palsson, B. O. The Escherichia coli MG1655 in silico metabolic genotype: its definition, characteristics, and capabilities. *Proc Natl Acad Sci U S A* **97**, 5528–5533 (2000).

[43] Edwards, J. S., Ibarra, R. U. & Palsson, B. O. In silico predictions of Escherichia coli metabolic capabilities are consistent with experimental data. *Nat Biotechnol* **19**, 125–130 (2001). DOI 10.1038/84379.

[44] Feist, A. M. *et al.* A genome-scale metabolic reconstruction for escherichia coli k-12 mg1655 that accounts for 1260 orfs and thermodynamic information. *Mol Syst Biol* **3**, 121 (2007). DOI 10.1038/msb4100155.

[45] Sumner, L. W., Journet, E. P. & Ulrike, M. (eds.). *The Medicago truncatula handbook*, chap. Growing *M. truncatula*: choice of substrates and growth conditions, 1–26 (The Medicago Consortium, 2006).

[46] Lisec, J., Schauer, N., Kopka, J., Willmitzer, L. & Fernie, A. R. Gas chromatography mass spectrometry-based metabolite profiling in plants. *Nat Protoc* **1**, 387–396 (2006). DOI 10.1038/nprot.2006.59.

[47] Hara, A. & Radin, N. S. Lipid extraction of tissues with a low-toxicity solvent. *Anal. Biochem.* **90**, 420–426 (1978).

[48] Williams, T. C. R. *et al.* Metabolic network fluxes in heterotrophic arabidopsis cells: stability of the flux distribution under different oxygenation conditions. *Plant Physiol* **148**, 704–718 (2008). DOI 10.1104/pp.108.125195.

[49] Sambrook, J. & Russel, D. *Molecular Cloning - A Laboratory Manual* (Cold Spring Harbour Laboratory Press, 2000).

[50] Antoniewicz, M. R., Kelleher, J. K. & Stephanopoulos, G. Accurate assessment of amino acid mass isotopomer distributions for metabolic flux analysis. *Anal. Chem.* **79**, 7554–7559 (2007).
